# Surgical Management of Distal Tibia Fracture: Towards An Outcome-based Treatment Algorithm

**DOI:** 10.5704/MOJ.2011.010

**Published:** 2020-11

**Authors:** I Rushdi, A Che-Ahmad, KAH Abdul-Ghani, R Mohd-Rus

**Affiliations:** 1 Department of Orthopaedics, International Islamic University Malaysia, Kuantan, Malaysia; 2 Department of Orthopaedics, Tengku Ampuan Rahimah Hospital, Kuantan, Malaysia; 3 Department of Community Medicine, International Islamic University Malaysia, Kuantan, Malaysia

**Keywords:** distal tibia fracture, tibia pilon fracture, tibial plafond fracture, Ilizarov external fixator, internal fixation

## Abstract

**Introduction:**

Distal tibia fractures are frequently associated with an extensive soft tissue injury which then leads to a higher risk of complications such as infection, non-union and eventually poor overall outcome. The purpose of this study is to measure the outcome of distal tibia fractures treated with internal fixation, external fixator or Ilizarov external fixator(IEF). We aim to propose an algorithm for management of distal tibia fractures by evaluating the treatment options, outcomes and risk factors present.

**Material and Methods:**

This study is a cross-sectional study of all distal tibia fractures treated surgically in Tengku Ampuan Rahimah Hospital, Klang from 1st January 2016 till 30th June 2018. Patient records were reviewed to analyse the outcomes of surgical treatment and risk factors associated with it.

**Results:**

Ninety-one patients were included with a mean age of 41.5 years (SD = 16.4). Thirty-nine cases (42.9%) were open fractures. Thirty-eight patients (41.8%) were treated with internal fixation, 27 patients (29.7%) were treated with IEF and 26 patients (28.6%) were treated with an external fixator. Among open fractures cases, no significant finding can be concluded when comparing each surgical option and its outcome, although one option was seen better than the other in a particular outcome. Initial skeletal traction or temporary spanning external fixator in close fractures reduced the risk of mal-alignment (p value=0.001). Internal fixation is seen superior to IEF and external fixator in close fractures in term of articular surface reduction (p value = 0.043) and risk of mal-alignment (p value = 0.007).

**Conclusion:**

There is no single method of fixation that is ideal for all pilon fractures and suitable for all patients. This proposed algorithm can help surgeons in deciding treatment strategies in the challenging management of distal tibia fractures to reduce associated complications.

## Introduction

Distal tibia fracture is a fracture that involves the metaphyseal area of the distal tibia and may extend to its weight-bearing articular surface^1^. It is also known as tibial pilon fracture or tibial plafond fracture if it involves the articular surface. Etienne Destot introduced the term tibial pilon in 1991 where pilon is a French word for pharmacist’s pestle that has a similar shape to the area of distal tibia metaphysis extending 5cm from ankle joint^2^. Plafond also comes from a French word that means ceiling which describes the horizontal articular surface of the distal tibia^2^.

The incidence of distal tibia fracture ranged from as low as 3 per 10,000 per year to as high as 28 per 10,000 per year depending on age and gender^3^. Pilon fractures are rare. They account for 1% of all lower limb fractures, 3% to 10% of all fractures of the tibia1 and approximately 20% to 40% are open fractures^4^. These fractures are usually associated with high energy trauma, caused by fall from heights or motor vehicle accidents thus they are frequently associated with extensive soft tissue injury and are often open fractures. These associations lead to a higher risk of infection, malunion, non-union and eventually poor overall outcome.

Distal tibia fracture is classified using the Arbeitsgemeinschaft für Osteosynthesefragen/Orthopaedic Trauma Association AO/OTA 43 classification 2018, which divides it into A, B and C. 43.A is extra-articular with subtypes A1 (simple), A2 (wedge) and A3 (multifragmentary). 43.B is partial articular with subtypes B1 (split fracture), B2 (split-depression fracture) and B3 (depression fracture). Meanwhile, 43.C is complete articular with subtypes C1 (simple articular, simple metaphyseal fracture), C2 (simple articular, multifragmentary metaphyseal fracture) and C3 (multifragmentary articular and metaphyseal fracture)^5^. Additionally, intra-articular distal tibia pilon fracture is categorised into three types by Ruedi and Allgower depending on articular surface dislocation and fracture comminution^6^.

Distal tibia fracture can be treated with a wide range of treatment methods including a variety of external fixators, intramedullary nailing and internal plate fixation. Minimally invasive techniques have been preferred recently with the hope of better outcomes^7-9^.

Historically, distal end tibia fractures were treated conservatively with traction followed by early range of motion. This approach was based on the concept of ligamentotaxis where soft tissue attachment to the bone will reduce the fractures but then it was realised that there was no soft tissue attachment to reduce the fractures in a severely comminuted fracture^10^.

Later on, open reduction and internal fixation became more accepted after publications by Ruedi and Allgower and research by the AO group. In their publication, Ruedi and Allgower developed a reproducible technique and stated fundamental operative principles for the management of intra-articular distal tibia fracture with 70 percent of their cases showing good or excellent late results^6,11^. However, other authors were unable to reproduce the result.

Studies on distal tibia fractures showed a variety of results but none can demonstrate that one is specifically better than the other for every type of this fracture. Hence, it is challenging for a surgeon to decide what is best for a patient considering other presenting factors which may increase the risk of complications.

The purpose of this study was to measure the outcome of distal tibia fractures treated with internal fixation, external fixator or Ilizarov external fixator. The study also aimed to identify the complications following surgically treated distal tibia fractures and to identify risk factors associated with the outcomes. Finally, we would like to propose an algorithm for the management of distal tibia fractures.

## Materials and Methods

This cross-sectional study included all distal tibia fractures that were treated surgically with any form of internal fixation, spanning external fixation or Ilizarov external fixator (IEF) in Tengku Ampuan Rahimah Hospital, Klang, done between 1st January 2016 till 30th June 2018. After approval from Malaysia Medical Research and Ethics Committee (MREC) through the National Medical Research Register (NMRR) and approval from Kulliyyah of Medicine Research Committee of International Islamic University Malaysia, all skeletally matured patients who underwent distal tibia fixation and did not have other concomitant ipsilateral ankle injuries or pre-existing ankle deformity were identified from the surgical record database. Sample size was calculated based on the study done by A.D. Duckworth *et al*^12^ in 2016, taking patients with complex intra-articular fracture of distal tibia plafond who had primary open reduction internal fixation (ORIF) or primary external fixator with delayed ORIF that developed infection (17.6%) with precision of 0.05, the minimum sample size required is 223 (n=223). However, due to time and logistic constraint during the data collection period, only 96 patients were identified (giving a precision of 0.07-0.08). We reviewed each patient’s records and radiographs for their age; gender; co-morbidity; smoking history; mechanism of injury; type and classification of fracture; surgical treatment; waiting time; surgeon; post-operative radiological review in term of alignment and articular surface restitution; time of union; range of motion and complications which includes infections, union complications, ankle arthritis and amputation. The reviews were made at least six months after the definitive surgery.

A superficial infection or pin tract infection was defined as any sign of an infection that healed with or without antibiotics and by just wound care and dressing. Deep infection was defined as an infection that needs surgical debridement in operating theatre^12^. Osteomyelitis was defined as deep bone infection shown clinically or radiographically and confirmed by surgical findings intra-operatively. Delayed union was defined when the union was delayed more than 23 weeks^13^. Non-union was defined as a fracture that has not healed nine months after the operation and there is no visible progress of healing during the last three months^14^. Mal-alignment was described as when there is more than 5° of angulation in any plane^15^. Articular incongruency was noted when there was any articular step seen radiologically after surgical intervention. Ankle arthritis was identified when there was osteophytes formation, subchondral sclerosis with or without reduced joint space^16^.

In our centre, the surgical option of treatment was determined by the surgeon on-call or surgeon in charge of the patient’s respective ward. Upon presentation to the emergency department, all patients were managed using standard trauma resuscitation protocol. Open fractures were irrigated, covered with intravenous cefuroxime then sent for thorough wound debridement and joint bridging external fixator. Triangular frame cross ankle external fixator was usually employed with two Schanz pin over the tibia proximal to fracture and a Denham pin through the calcaneum connected with two bars. Associated lateral malleolus fracture was managed with a rush rod or intra-medullary wire. Near all external fixator cases were performed by registrars.

For closed fractures, first, the patient’s limb was put on a splint, elevated and regular cryotherapy was applied. Then, the patient was put on calcaneal traction or keep on the splint until definite surgery. Definitive treatment and time to surgery were decided depending on soft tissue condition and fracture configuration. In many open fracture cases, if the fracture reduced well with spanning external fixator, especially in poor soft tissue condition, the external fixator was kept as definitive management until soft callus formation before a decision was made to convert to cast if wound healing was permissible. Ilizarov external fixator (IEF) is generally opted for in cases of severe comminuted open fracture while open reduction, internal fixation (ORIF) is usually chosen in cases of closed fracture and simple extra-articular fracture.

All data were analysed using IBM SPSS version 23. The descriptive data were expressed as frequency with percentage as well as mean ± standard deviation unless otherwise stated. Data were cross-tabulated and evaluated statistically using Chi-square test or Fisher’s exact test. Association between outcome of surgical treatment of distal tibia fracture, types of treatment and risk factors were evaluated. A p-value of 0.05 or less was considered significant.

## Results

A total of 91 patients fulfilled the inclusion criteria during the data collection period. Five patients were lost to follow-up, therefore excluded. Due to limitations of our patient's data registry, any data that was not available was marked as missing data.

The mean age of the 91 patients was 41 years (15 to 81) whereby 68 (74.7%) were male. Of the 91 patients, 74 patients were involved in a motor vehicle accident (81.3%), 15 patients sustained their injury from a fall (16.5%), and the remaining two patients were involved in industrial injuries (2.2%). 39 cases (42.9%) were open fractures while the remaining 52 cases (57.1%) were closed fractures. 68 cases (74.7%) were extra-articular AO/OTA 43.A, 11 cases (12.1%) were partial articular 43.B, and 12 cases (13.2%) were complete articular 43.C. 38 patients (41.8%) were treated with internal fixation, 27 patients (29.7%) were treated with IEF and 26 patient (28.6%) were treated with external fixator (Table I).

**Table I:** Background characteristics of patients (n=91)

Variables	IF (n=38)	No. (%) IEF (N=27)	EF (N=26)
Age (mean±SD)	40.2±14.4	42.8±16.4	42.2±19.6
Gender			
Male	25(65.8)	22(81.5)	21(80.8)
Female	13(34.2)	5(18.5)	5(19.2)
Fracture type			
open	3(7.9)	15(55.6)	21(80.8)
close	35(92.1)	12(44.4)	5(19.2)
Fracture classification			
A	25(65.8)	21(77.8)	22(84.6)
B	7(18.4)	2(7.4)	2(7.7)
C	6(15.8)	4(14.8)	2(7.7)
Initial traction			
No	29(76.3)	7(26.9)	0(0)
Yes	9(23.7)	19(73.1)	26(100)
Smoking			
No	26(68.4)	20(74.1)	18(69.2)
Yes	12(31.6)	7(25.9)	8(30.8)
Diabetes			
No	30(78.9)	20(74.1)	22(84.6)
Yes	8(21.1)	7(25.9)	4(15.4)
Waiting time (days)			
median	11	17	1
Surgeon			
Registrar	9(23.7)	0(0)	25(96.2)
Specialist	23(60.5)	4(14.8)	1(3.8)
Consultant	6(15.8)	23(85.2)	0(0)

Ankle arthritis and mal-alignment were the highest complications seen both at 23.1%. Others were superficial infection (15.4%), deep infection (14.3%), delayed union (17.6%), non-union (12.1%) and 2 cases of amputation (Table II).

**Table II:** Complications of distal tibia fracture

Complications	No (%)
Superficial / pin site infection	14 (15.4)
Deep infection / osteomyelitis	13 (14.3)
Amputation	2 (2.2)
Delayed union	16 (17.6)
Non-union	11 (12.1)
Mal-alignment	21 (23.1)
Articular incongruency	5 (4.4)
Ankle arthritis	21 (23.1)

*more than 1 complications may occur in one patient.

The implant used for internal fixations varies from screw fixation, medial or anterolateral distal tibia locking plate of different company systems. Surgeons who performed the surgery were at least senior registrars or trainees while comminuted intra-articular fractures were reserved specifically for senior specialists. 60.5% of internal fixator cases done by specialists, 23.7% cases done by senior registrar and only 15.8% cases done by consultants. IEF cases were performed by trained specialists who are advanced trauma specialists only. Three full rings with foot and ankle frame extension were usually employed in these cases. Full rings were connected with 3 rods. Two proximal tibia rings were fixed with 2 Schanz pins or one pin and two tensioned wired. The distal tibia ring was fixed with two tensioned wires, sometimes with Schanz pin, attached to the reconstructed distal tibia fragments.

Among open extra-articular fracture cases, less incidence of infection seen in patients treated with IEF and external fixator group when compared to internal fixation. One case of open fracture treated with internal fixation complicated with deep infection (100%). Compared to patients treated with IEF and external fixator that was not complicated with infection with seven (53.8%) and nine (64.3%) each group.

Then, when comparing external fixator and IEF, external fixator cases were seen to have a higher risk of mal-alignment. Patients with an open extra-articular fracture that were treated with external fixators had a 55.6% risk of mal-alignment when compared to IEF with 28.6% risk (Table III). Still, putting on skeletal traction or temporary external fixator before definitive fixation in open fracture cases lowed the risk of infection and risk of alignment deformity. Open fracture cases that were not on skeletal traction or temporary external fixator were complicated with 100% risk of infection and 50% risk mal-alignment compared to patients with skeletal traction or temporary external fixator at 43.3% risk of infection and 38.9% risk of mal-alignment (Table IV).

**Table III:** Outcome of distal tibia fracture treated with internal fixation (IF), Ilizarov external fixator (IEF) or external fixator (EF) (n=91)

	N (%)															
	Open fracture (n=39)								Close fracture (n=52)							
	Extra-articular (n=35)			p-value	Intra-articular (n= 4)			p-value	Extra-articular (n=33)			p-value	Intra-articular (n=19)			p-value
	IF (n=2)	IEF (n=14)	EF (n=19)		IF (n=1)	IEF (n=1)	EF (n=2)		IF (n=23)	IEF (n=7)	EF (n=3)		IF (n=12)	IEF (n=5)	EF (n=2)	
Infection																
No	0	7 (53.8)	9 (64.3)	0.234	0	1 (100)	0	0.999	15 (68.2)	6 (85.7)	1 (50.0)	0.451	7 (70.0)	4 (80.0)	2 (100)	0.999
Superficial	0	4 (30.8)	1 (7.1)		1 (100)	0	1 (100)		2 (9.1)	0	1 (50.0)		3 (30.0)	1 (20.0)	0	
Deep	1 (100)	2 (15.4)	4 (28.6)		0	0	0		5 (22.7)	1 (14.3)	0		0	0	0	
Malignment																
No	2 (100)	10 (71.4)	8 (44.4)	0.17	1 (100)	1 (100)	1 (50.0)	0.999	23 (100)	5 (71.4)	1 (33.3)	0.003	11 (100)	5 (100)	0	0.007
Yes	0	4 (28.6)	10 (55.6)		0	0	1 (50.0)		0	2 (28.6)	2 (66.7)		0	0	2 (100)	
Union																
United	1 (100)	6 (54.5)	6 (50.0)	0.999	1 (100)	1 (100)	0	0.999	15 (71.4)	3 (42.9)	1 (50.0)	0.158	8 (80.0)	4 (80.0)	1 (50.0)	0.811
Delayed	0	3 (27.3)	3 (25.0)		0	0	0		5 (23.8)	2 (28.6)	0		1 (10.0)	1 (20.0)	1 (50.0)	
Non-union	0	2 (18.2)	3 (25.0)		0	0	1 (100)		1 (4.8)	2 (28.6)	1 (50.0)		1 (10.0)	0	0	
Articular																
Incongruency																
No	NA				1 (100)	1 (100)	1 (50.0)	0.999	NA				10 (90.9)	1 (25.0)	2 (100)	0.043
Yes					0	0	1 (50.0)						1 (9.1)	3 (75.0)	0	
Ankle Arthritis																
No	0	7 (77.8)	6 (54.5)	0.472	1 (100)	1 (100)	1 (100)	0.999	19 (90.5)	5 (71.4)	1 (50.0)	0.118	8 (80.0)	1 (20.0)	1 (50.0)	0.115
mild	0	2 (22.2)	1 (9.1)		0	0	0		2 (9.5)	1 (14.3)	1 (50.0)		1 (10.0)	1 (20.0)	1 (50.0)	
severe	1 (100)	1 (11.1)	4 (36.4)		0	0	0		0	1 (14.3)	0		1 (10.0)	3 (60.0)	0	

**Table IV:** Comparison of outcome with or without skeletal traction or external fixator (n=91)

	n (%)					
	No traction	Open fracture With traction	p-value	No traction	Close fracture With traction	p-value
Infection						
No	0	17 (56.7)		22 (71.0)	13 (81.3)	
Superficial/pin tract	1 (100)	6 (20.0)	0.453	5 (16.1)	2 (12.5)	0.883
Deep	0	7 (23.3)		4 (12.9)	1 (6.3)	
Mal-alignment						
No	1 (50.0)	22 (61.1)	0.64	33 (100)	11 (64.7)	0.001
Yes	1 (50.0)	14 (38.9)		0	6 (35.3)	
Articular incongruency (intra-articular type only)						
No	0	3 (75.0)	NA	9 (81.8)	4 (66.7)	0.445
Yes	0	1 (25.0)		2 (18.2)	2 (33.3)	

Nevertheless, patients treated with IEF had a higher risk of delayed or non-union compared to those that were treated with internal fixation. Open fractures that were treated with internal fixation achieved union on time while those on IEF were complicated with either delayed union (27.3%) or nonunion (18.2%) (Table III).

Among closed extra-articular fractures, internal fixation had a higher risk of infection whereby 68.2% of cases where not complicated with infection when compared to 85.7% in IEF cases. Internal fixation also associated with a lower risk of mal-alignment in close fractures. No mal-alignment was seen in close extra-articular fractures treated with internal fixation when compared to 28.6% mal-alignment in IEF and 66.7% in external fixator (p value = 0.003). In close extra-articular fractures, internal fixation had a lower risk of delayed or non-union compared to IEF or external fixator; 71.4% of closed extra-articular fractures that were treated with internal fixation united in time as compared to 42.9% of patient treated with IEF and 50.0% patient treated with external fixator (Table III).

Meanwhile, in close intra-articular fracture, no infection where seen in 70% of cases treated with internal fixation when compared to 80% with IEF and 100% with external fixator. Similar to the significant finding in close extra-articular fracture, no mal-alignment was seen in close intra-articular fracture treated with internal fixation or IEF when compared to 100% risk of mal-alignment in patient treated with external fixator (p-value 0.007). Both internal fixation and IEF had a similar risk of delayed or non-union with 80% of both groups united in the expected time. In terms of articular congruency, internal fixation was associated with better articular surface reduction when compared to IEF (p value = 0.043). 90.9% of closed intra-articular fractures treated with internal fixation had no articular step compared to 25% in the IEF group, although good articular surface reduction was also seen in external fixator (Table III).

Initial skeletal traction had significant effect on reducing risk of mal-alignment in close fractures (p value = 0.001). No significant effect of initial traction was seen on the risk of infection or articular surface reduction (Table IV).

The occurrence of ankle arthritis was seen more frequently in patients treated with IEF or external fixator as compared to patients treated with internal fixation in close fracture. However, this finding was not seen in open fractures (Table III).

Other findings included a mean union time of 25 weeks (SD =10) seen in close extra-articular fractures, and that the time to definitive surgery for closed fractures treated with internal fixation was not a significant risk factor for infection. Closed fractures that were treated with internal fixation and which were not complicated with infection were mostly were operated on between 3-29 days (median 11 days) post-trauma. This overlapped with time to the definitive procedure for cases complicated with infection that were operated between 7-16 days (median 13 days) post-trauma.

Non-smoker patients had a slightly better union at 68.0% when compared to smoker patients at 54.2%. Non-diabetic patients also have a better union rate and a somewhat lower risk of infection (Table V).

**Table V:** Union complication and infections stratified to smoker vs non-smoker and diabetic vs non-diabetic (N=91)

	n (%)					
	Smoker	Non-smoker	p-value	Diabetic	Non-diabetic	p-value
Union						
United	13 (54.2)	34 (68.0)	0.294	10 (55.6)	37 (66.1)	0.575
Delayed	8 (33.3)	8 (16.0)		4 (22.2)	12 (21.4)	
Non-union	3 (12.5)	8 (16.0)		4 (22.2)	7 (12.5)	
Infection						
No	18 (69.2)	34 (54.2)	0.802	11 (61.1)	41 (67.2)	0.778
yYes	8 (30.7)	19 (35.9)		7 (38.9)	20 (32.8)	

## Discussion

Following the recommendation by Rüedi and Allgöwer, many studies were conducted on distal tibia fracture, particularly on intra-articular pilon fracture. These studies showed interesting results and some recommended new concepts of management. McFerran *et al* stated that surgical treatment of this type of fracture was associated with high risk of complications^17^. Watson *et al* found that the complication rates were higher in the open plating group when compared to the external fixator group^18^. They both emphasised on better soft tissue management apart from solely concentrating on bone management. Sirkin *et al* also recognised the importance of better appreciation of soft tissue to reduce complications in pilon fracture^19^. He advocated staged treatment protocol to allow soft tissue stabilisation before open reduction and internal fixation. Recently in 2016, Duckworth *et al* studied the outcome of pilon fractures following operative intervention^12^. He reported a satisfactory outcome for early primary open reduction and internal fixation in most patients and a higher rate of overall infection in the staged protocol of primary external fixation with delayed open reduction and internal fixation.

Comparative studies on this type of fracture had conflicting results. One study reported a higher risk of infection in ORIF compared to external fixation. Lower mean clinical scores and other outcomes were observed for ORIF although not statistically significant. More complications were also seen in patients treated with ORIF^20^. Another study showed excellent or good objective and subjective results in dynamic external fixation but noted high rates of superficial infections and arthrosis^21^. ORIF also was noted to has 2% rate of amputation, 2% rate of arthritis, 2% rate of chronic osteomyelitis drainage, 2% rate of wound dehiscence and 13% rate of skin necrosis^19^.

Other comparative studies on ORIF versus external fixator showed that patients treated with external fixation had more complications than ORIF, and ORIF had a higher union rate^22,23^. Lower clinical scores and more loss of range of motion were also seen in the external fixator group^22^. While, two-staged external fixator and plate fixation, rates of infection and arthrodesis were reported lower compared to primary ORIF or single staged procedure^24^.

**Fig. 1:**
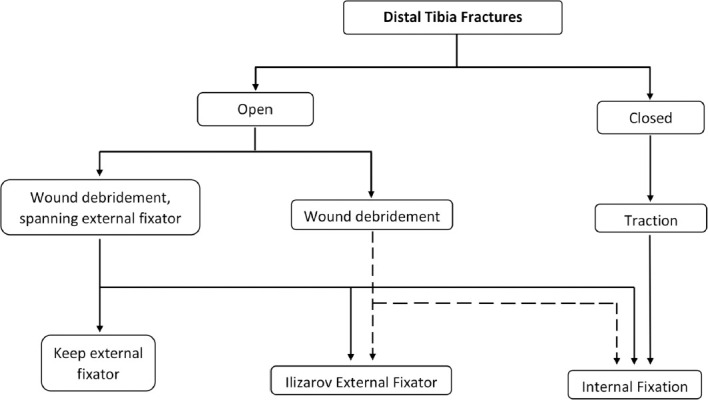
Proposed algorithm for management of distal end tibia fracture.

In our study, we divided each type of fracture according to the type and articular involvement. Then, we observed the risk of complications and the outcome of each type of treatment in each group. By comparing the risk of complications of each treatment method, we built the algorithm of management of distal tibia fracture as shown in Fig. 1.

Among open fracture cases, when comparing each surgical option and its outcome, no significant finding can be concluded although one type of surgical option was seen better than the other in a particular outcome. As per standard management of open fracture^25^, urgent wound debridement is a must. In our observation, initial skeletal traction or spanning external fixator do not have a significant effect on the risk of infection, mal-alignment or articular incongruency. Spanning external fixator can be applied during initial wound debridement and can be kept definitively till fracture union or converted to internal fixation if the wound is clean or to Ilizarov external fixator if otherwise. Surgeon can also choose to keep the patient limb on slab or splint after wound debridement and perform IEF or internal fixation later when condition permissible.

Nearly all open fractures were treated with either spanning external fixator or IEF but there were two patients with open extra-articular fracture that were treated with internal fixation. One of them is a high grade open fracture that where treated initially with external fixator. He was treated with medial distal tibia locking plate but later on complicated with deep infection and ankle arthritis. Fortunately, the infection settled with wound debridement and the fracture united at 12 weeks after internal fixation. The other case of open extra-articular fracture was a low grade open fracture, that was treated with screw fixation. Early follow-up showed good alignment but the subject was lost to follow-up and we were unable to measure other outcomes. The other case was an open intra-articular fracture, treated with medial distal tibia locking plate after five days of wound debridement and skeletal traction. Although the case was complicated with superficial infection, the fracture united well and no other complication occur.

For close fractures, we recommend putting on skeletal traction or temporary spanning external fixator as first line of treatment as this has been shown to reduce the risk of mal-alignment. Internal fixation is seen superior to IEF and external fixator in close fractures in terms of articular surface reduction and risk of mal-alignment. Although not statistically significant, in close fractures, internal fixation has a better union rate and lower risk of ankle arthritis but a slightly higher risk of infection.

Nevertheless, we would not advocate internal fixation for closed fractures with poor soft tissue condition such as blisters and superficial wounds. A severely comminuted fracture that is not amendable to hold with plate and screws should not proceed for internal fixation.

Comparing the results of our study to other previous studies, there are both similar and conflicting results. Our proposed algorithm differs in few aspects. First, we do not advocate for all patient to be put on skeletal traction or spanning external fixator as initial temporary measure before definitive fixation. Secondly, we believe spanning external fixator has its role to be a definitive fixation in selective cases. Lastly, we suggest internal fixation as a preferred method of fixation for close fracture over IEF. Having said that, our study is limited by inadequate patient record systems, multiple surgeons involvement, unequal distribution within the group, study design, small sample size and short study period. Nonetheless, previous studies also were unable to produce specific evidence due to biases and other confounding factors presenting with this type of fracture.

For future studies, it is worth including intramedullary nail which is another recommended method of treatment that has gained much attention in recent years, especially for type 43A in view of its minimally invasive approach.

## Conclusion

In conclusion, there is no single method of fixation that is ideal for all pilon fractures and suitable for all patients. This proposed algorithm can help surgeons in deciding the strategy of treatment while considering other associated factors. The fracture pattern, soft-tissue condition, patient comorbidities, surgical skills and experience as well as hospital resources must always be taken into account. However, further studies are needed to prove the effectiveness of this algorithm.
